# Hospitalization and mortality following non-attendance for hemodialysis according to dialysis day of the week: a European cohort study

**DOI:** 10.1186/s12882-020-01874-x

**Published:** 2020-06-09

**Authors:** James Fotheringham, Michael T. Smith, Marc Froissart, Florian Kronenberg, Peter Stenvinkel, Jürgen Floege, Kai-Uwe Eckardt, David C. Wheeler

**Affiliations:** 1 Northern General Hospital, Sheffield Kidney Institute, Herries Road, Sheffield, South Yorkshire S5 7AU UK; 2 School of Health and Related Research, University of Sheffield, Sheffield, UK; 3 Computer Science, University of Sheffield, Sheffield, UK; 4 Clinical Trial Unit, Centre Hospitalier Universitaire Vaudois, Lausanne, Switzerland; 5 Division of Genetic Epidemiology, Medical University of Innsbruck, Innsbruck, Austria; 6 Department of Renal Medicine, Karolinska University Hospital Huddinge, Stockholm, Sweden; 7 Department of Nephrology, RWTH University of Aachen, Aachen, Germany; 8 Department of Nephrology and Medical Intensive Care, Charité – Universitätsmedizin-Berlin, Berlin, Germany; 9 Department of Renal Medicine, University College London, London, UK

**Keywords:** Hemodialysis, Adherence, Compliance, Interdialytic interval, Hospitalisation, Mortality

## Abstract

**Background:**

The extension of the interdialytic interval due to due to dialysis session non-attendance varies according to which session of the week the patient misses. The impact of this on subsequent hospitalization and mortality is unknown.

**Methods:**

The ARO cohort study prospectively collected data from hemodialysis patients across 15 European countries on demography, comorbidity, laboratory, hospitalisation, mortality and individual hemodialysis sessions from 2007 to 2014. Event rates for death and hospitalisation according to dialysis day of the week were calculated for patients who attended the three previous scheduled hemodialysis sessions, who then on the next scheduled dialysis day either attended or did not attend. The hazard ratio for these events following non-attendance for the first compared to the second dialysis session of the week was estimated using Cox proportional hazards model adjusted for patient demographics.

**Results:**

3.8 million hemodialysis sessions in 9397 patients were analysed. The non-attendance rates for Monday/Wednesday/Friday sessions were 0.8, 0.9% & 1.4% respectively, and for Tuesday/Thursday/Saturday sessions were 0.6, 1.0% & 1.2% respectively. Compared to those who attended, for the 48–72 h between non-attendance and the next scheduled haemodialysis session, mortality significantly increased from 4.86 to 51.9/100 pt-yrs and hospitalisation increased from 0.58 to 2.1/yr. As time from the two-day break increased, the risk associated with non-attendance lessened: compared to missing the second hemodialysis session, missing the first session had a hazard ratio for mortality of 2.04 (95% CI 1.27–3.29), and for hospitalisation 1.78 (95% CI 1.29–2.47). In patients who attended their scheduled dialysis session and the three preceding, after the two-day break there were absolute increases in mortality (8.3 vs. 4.9/100 pt-yrs) and hospitalisation (1.0 vs. 0.6/yr for the rest of the week) comparable to previous studies.

**Conclusions:**

In addition to hospitalisation and mortality increases seen after the two-day break, additional harm may be manifested in the greater increases in mortality and hospitalisation observed after non-attendance for the first hemodialysis session after the two-day break compared to missing other sessions.

## Background

Non-attendance for dialysis is common, with the proportion of sessions affected between 0.6% in Europe and Japan to 7.9% in the United States [1]. Non-adherence is often assumed to reflect underlying psychological factors [2], but the evolution of acute illness, complications with dialysis access, rescheduling for planned admissions and holidays also lead to changes in the dialysis schedule [3]. These mechanisms may be a partial explanation for the association between non-attendance and hazard ratios for hospitalisation of 1.13 to 3.98 and medium-term mortality of 1.33 to 2.36 [1, 4–6]. However, all but one of these studies recruited patients after the non-adherent session of interest and/or analysed the occurrence of endpoints months to years afterwards, thereby reflecting the risk of non-adherent phenotype rather than the short-term risk of skipping a HD session.

Over 95 % of patients worldwide receiving (HD) for treatment of kidney failure are offered three dialysis sessions per week, with the most commonly assigned schedules being Monday/Wednesday/Friday (MWF) and Tuesday/Thursday/Saturday (TTS) [7]. The HD session before the two day break usually falls before a weekend on a Friday or Saturday, when patients are likely to engage in activities which they may prioritise over attending for dialysis. Non-attendance for the first HD session of the week means that the immediate period after non-attendance is associated with the highest exposure to fluid, potassium and uraemic toxins, and non-attendance for the third HD session of the dialysis week potentially results in the extension of the two-day break to a four-day break. The variation in the harm caused by non-attendance in relation to the length of the preceding interdialytic interval is not known. Furthermore, to date the large studies that have explored the harm associated with the “long gap” have used a range of approaches to determine the patient’s HD schedule [8–10] and have not ensured patients reliably attended. To explore these issues, we examined data from a large European cohort of in-centre HD patients on three times-a-week schedules [11] who attended their scheduled dialysis fully.

## Methods

This study explored the hypothesis that there may be differential risk of mortality and hospitalisation following non-attendance according to the interdialytic interval.

### Study population

The Analyzing Data, Recognizing Excellence and Optimizing Outcomes (ARO) cohort study contains anonymised longitudinal individual-level data for patients starting HD who were enrolled at one of the 312 Fresenius Medical Care (FMC) facilities across 15 European countries between 2007 and 2009 and followed up until the end of 2014. Data on demography, comorbidity, laboratory, hospitalisation, mortality and individual HD sessions were captured prospectively [12]. The study cohort used for this analysis was limited to patients who were prescribed three times a week HD, who had survived more than 90 days after starting HD, had HD session and laboratory data available at any time-point during the study, and in whom a MWF or TTS schedule could be identified. They were censored following transplantation, change in dialysis modality, transfer to a non-FMC facility or lost to follow-up.

### Hemodialysis schedule, non-attendance, hospitalisation and mortality

In patients who were prescribed three times a week HD, for each day of follow-up time we reviewed the preceding seven days to identify on which days HD was conducted, enabling us to identify MWF and TTS schedules. We did not analyse patients receiving the less common Tuesday/Thursday/Sunday schedule, as TTS patients could miss a Saturday session, and be offered a Sunday session instead. This would be interpreted as a Tuesday/Thursday/Sunday schedule and would artificially lower the rate of non-attendance on Saturday in TTS patients. The first HD session after a scheduled long interval was defined as the first day of the dialysis week, this being Monday for patients dialysing MWF and Tuesday for patients dialysing TTS.

We examined each scheduled dialysis day, first confirming that the three preceding scheduled MWF or TTS sessions had been attended. We then assessed for non-attendance determined by the absence of a recorded pre-dialysis blood-pressure, pre-dialysis weight and dialysis treatment time. From these we excluded dialysis days and associated events in patients who were hospitalized or died on the identified day of non-attendance as these events which prevent attendance would not represent non-adherence. We also excluded patients who had been discharged from hospital in the preceding seven days and those who had an un-scheduled HD session on the preceding day potentially resulting in a temporary or permanent change to their dialysis schedule. Patients who had missed an HD session but then return to a MWF or TTS pattern then re-entered the data set from this point. A record of hospital admission is made by the clinic staff and those are required when a patient missed at least one treatment. The analysis was limited to hospitalizations lasting longer than one night, which do not involve routine dialysis vascular access care (International Classification of Disease Version 10 Codes pertaining to: adjustment and management of vascular access device, arteriovenous fistula, mechanical complication of vascular dialysis catheter, mechanical complication of other vascular grafts, other complications of cardiac and vascular prosthetic devices, implants and grafts, preparatory care for dialysis).

### Statistical analysis

Although all follow-up time on 3xW HD was considered in our analysis, in order to facilitate comparisons to other studies of non-adherence, the demographics of the cohort are reported stratified by whether the patient did not attend a HD session during the first 4 months of the study. We report the crude non-attendance as a proportion, the numerator being the number of missed scheduled HD sessions in non-hospitalised patients, who had an established MWF or TTS schedule and who have attended all three preceding scheduled HD sessions. The denominator was the number of scheduled HD sessions in the above patients and setting. These crude proportions are reported across dialysis days and between schedules, using multivariate logistic regression to determine if significant variation existed.

**Fig. 1:**
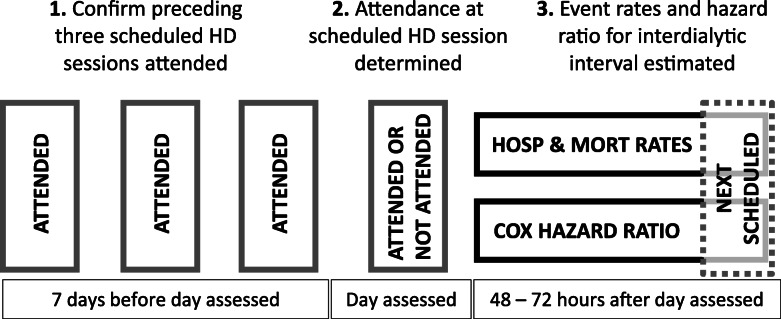
Illustration of the identification of complete attendance for three scheduled hemodialysis sessions, non-attendance, and subsequent mortality and hospitalisation

In order to quantify the per-day rates of hospitalisation and mortality and their relationship to the dialysis schedule we calculated hospitalisation (per patient year) and mortality (per 100 patient years) event rates for each individual dialysis day in individuals who attended the three preceding scheduled dialysis. These events are reported separately for the two dialysis schedules day and if the patient attended or did not attend the next scheduled dialysis session. For patients classified as not attending this HD session we reported hospitalisation and mortality rates for each day between the day after the missed session and the day of the next scheduled HD session (2 to 3 days), as shown in Fig. 1. Hospitalisation and mortality rates are effectively zero on the day of non-attendance due to our exclusion criteria. Event rates and associated confidence intervals around mortality and hospitalisation rates were estimated using the Poisson distribution, calculated by dividing the total number of events divided by the total patient time over which these events occurred (stratified by dialysis day and attendance). Although this does not fully capture within- and between-patient variation, we demonstrate that this is statistically appropriate in our supplementary materials. We fitted multivariate time-varying single event (mortality endpoints) and multiple event (hospitalization endpoints) Anderson and Gill Cox regression models [13]. In addition to being able to handle multiple events, The Anderson and Gill cox extension has been shown to be robust where discontinuous patient follow-up time is present (such as while a patient is hospitalized or during extended non-adherence) [14]. In patients who attended, this follow-up time was one day, allowing us to estimate the risk of hospitalisation for each individual day of the dialysis week. In those who did not attend, follow-up was counted from the day after the non-attended session until the next scheduled HD session (two or three days depending on the session missed, Fig. 1). All regression models used the second HD day (Wednesday or Thursday in MWF and TTS respectively) as a reference. In order to compare the risk of hospitalisation and mortality between attenders and non-attenders, we used the second dialysis day in those who attended as the reference day. All multivariate models were adjusted for age, gender, comorbidity (previous acute ischemic heart disease event, congestive cardiac failure, chronic obstructive pulmonary disease, cerebrovascular disease, diabetes, arrythmias, gastrointestinal bleeding, liver disease, other cardiac disease, peripheral vascular disease, cancer), time on dialysis and vascular access type. We did not adjust for dialysis session or laboratory variables as they are potentially in the causal pathway between non-attendance and our endpoints of interest [15]. We account for clustering of repeated observation and events in our hospitalisation analyses with a robust sandwich variance estimator.

The proportion of sessions fully attended prior to hospitalisation was estimated to determine the proportion of patients deviating from their schedule prior to admission: the numerator being the number of hospitalisations in which patients deviated from their scheduled HD sessions prior to hospitalisation, the denominator being all hospitalisations in three times a week patients on established MWF or TTS schedules.

We conducted the following sensitivity analyses. To explore if rescheduled or emergency dialysis identified through an FMC provider altered the variation in outcomes according to dialysis day of the week, we censored at the time when rescheduled dialysis was delivered. In an attempt to identify facility-directed dialysis rescheduling (e.g. moving from MWF to TTS) as an explanation for variation in non-attendance, we excluded non-attended sessions which preceded a new three times a week schedule that persisted for two weeks. All analyses were conducted in R version 3.4.4.

## Results

**Fig. 2:**
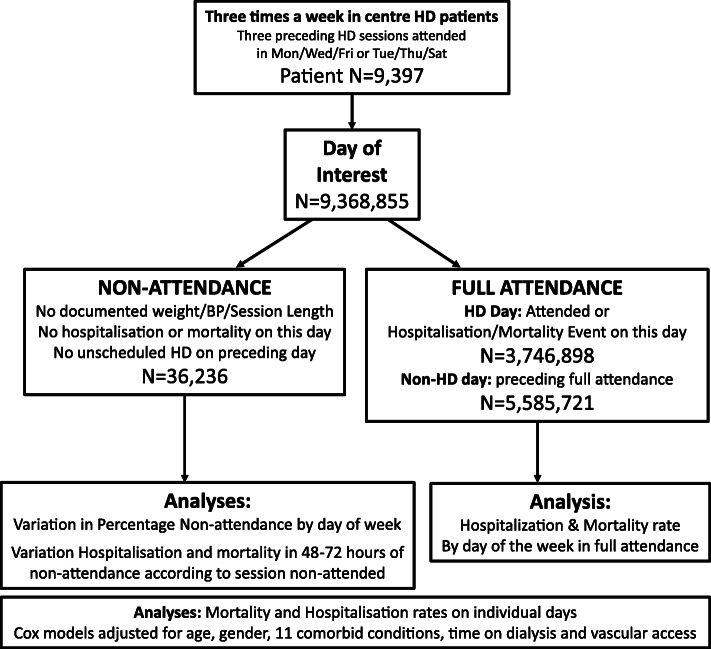
Summary of the number of patients, follow-up time, dialysis sessions and analytical methods employed

From the 11,211 patients in the ARO cohort, 9397 were identified as receiving three times a week HD, could be assigned a MWF or TTS schedule, had complete set of covariate data and had survived 90 days from entry into the study. The overall mortality rate was 11.0 deaths per 100 patient years, and the emergency hospitalisation rate was 0.66 per patient year. Figure 2 summarises the analysis of the 3.8 million HD sessions with full attendance (all three scheduled haemodialysis sessions prior to the day of analysis), representing 1.34 million weeks of treatment. There were 1376 deaths and 15,317 hospitalisations in the follow-up period following full attendance.

### Dialysis session non-attendance by schedule and day of the week

Following three consecutive attended HD sessions, the proportion of scheduled HD sessions that were not attended was 1.0% (36,236 of 3,783,134 sessions analysed). The characteristics of patients who attended compared to those did not attend one or more sessions during the first four months of the study are detailed in Table 1 and highlight that younger, male patients with less comorbidity were more likely not to attend a HD session.

**Table 1:** Patient characteristics according to non-attendance in the first 4 months of the study

	Attended (83.3%)	Non-Attended (16.7%)	P
n	7467	1501	
Age (mean (sd))	64.83 (14.72)	61.68 (15.16)	< 0.001
Interdialytic weight gain (Kg)	1.58 (1.06)	1.56 (1.10)	0.367
Systolic Blood Pressure (mmHg)	137.79 (24.15)	137.55 (24.72)	0.722
Diastolic Blood Pressure (mmHg)	71.34 (13.91)	73.43 (14.00)	< 0.001
Male (%)	4532 (60.7)	881 (58.7)	0.157
Dialysis Catheter	3408 (45.6)	743 (49.5)	0.007
Acute ischemic cardiac event	1107 (14.8)	199 (13.3)	0.126
Cancer	569 (7.6)	105 (7.0)	0.433
Cardiac Failure	1111 (14.9)	185 (12.3)	0.011
Chronic Obstructive Pulmonary Disease	461 (6.2)	60 (4.0)	0.001
Cerebrovascular Accident	672 (9.0)	93 (6.2)	< 0.001
Depression	112 (1.5)	21 (1.4)	0.859
Diabetes	2581 (34.6)	509 (33.9)	0.647
Dysarrythmia	672 (9.0)	98 (6.5)	0.002
Gastrointestinal Bleeding	98 (1.3)	19 (1.3)	0.984
Liver Disease	197 (2.6)	38 (2.5)	0.883
Other Cardiac Disease	51 (0.7)	10 (0.7)	1.000
Peripheral Vascular Disease	808 (10.8)	143 (9.5)	0.150

429 patients (4.6%) excluded from table due to missing blood pressure and weight data during the first 4 months of the study

A higher proportion of the third HD session were missed in both MWF and TTS schedules (Table 2). Compared to the middle dialysis day, the odds of missing the first and third dialysis days in MWF patients were 0.83 (95% CI 0.80–0.86) and 1.55 (95% CI 1.51–1.61) respectively. In TTS patients these were 0.63 (95% 0.61–0.67) and 1.28 (95% CI 1.24–1.34). Overall the proportion of sessions that were non-attended was similar across countries, but some variation in which session of the dialysis week was most commonly non-attended was evident (Supplementary Figure 1 in Additional File 1). In these patients scheduled to receive dialysis three times a week, 20.4% of emergency hospitalisations were preceded by a deviation from their three times a week schedule (3776/18,546) with no significant difference between MWF and TTS regimens (20.7 and 20.1% respectively, *P* = 0.32).

**Table 2:** Mortality and hospitalisation events and associated rates, stratified by hemodialysis attendance. Events following non-attended sessions are reflected in the columns of subsequent haemodialysis days (e.g. missing Monday’s HD session influences Tuesday and Wednesday’s events)

**Mon/Wed/Fri Schedule**	**Mon**	Tue	**Wed**	Thu	**Fri**	Sat	Sun
Non-Attendance rate (%)	0.77	NA	0.96	NA	1.44	NA	NA
Attended Admissions (n)	1866	1314	1409	956	1116	432	557
Attended Admit Rate (annualised)	0.94 (0.90–0.99)	0.66 (0.63–0.70)	0.71 (0.68–0.75)	0.48 (0.45–0.51)	0.57 (0.54–0.60)	0.22 (0.20–0.24)	0.28 (0.26–0.30)
Attended deaths (n)	150	70	111	82	95	67	78
Attended Death Rate (/100 pt. yrs)	7.6 (6.43–8.91)	3.52 (2.74–4.45)	5.63 (4.63–6.78)	4.12 (3.28–5.12)	4.84 (3.91–5.91)	3.39 (2.63–4.31)	3.9 (3.08–4.86)
Attended Days at risk	720,744	726,084	719,809	725,761	716,707	721,293	730,785
Non-Attended Admissions (n)	54	50	37	49	41	38	34
Non-Attended Admit Rate (annualised)	1.95 (1.47–2.54)	3.44 (2.56–4.54)	2.57 (1.81–3.54)	2.67 (1.97–3.53)	2.25 (1.61–3.05)	1.37 (0.97–1.89)	1.23 (0.85–1.72)
Non-Attended deaths (n)	11	13	8	12	8	10	3
Non-Attended Death Rate (/100 pt. yrs)	39.4 (19.7–70.6)	89 (47.4–152.3)	54.9 (23.7–108.3)	65.0 (33.6–113.5)	43.6 (18.8–85.8)	35.9 (17.2–65.9)	10.8 (2.2–31.5)
Non-attended Days at risk	10,107	5300	5264	6707	6655	10,096	10,087
**Tue/Thu/Sat Schedule**	Mon	**Tue**	Wed	**Thu**	Fri	**Sat**	Sun
Non-Attendance rate (%)	NA	0.58	NA	0.98	NA	1.18	NA
Attended Admissions (n)	1132	1638	1115	1207	781	822	425
Attended Admit Rate (annualised)	0.67 (0.63–0.71)	0.99 (0.94–1.04)	0.66 (0.62–0.7)	0.73 (0.69–0.77)	0.46 (0.43–0.5)	0.5 (0.46–0.53)	0.25 (0.23–0.28)
Attended deaths (n)	106	152	61	83	59	88	40
Attended Death Rate (/100 pt. yrs)	6.28 (5.14–7.6)	9.16 (7.76–10.74)	3.63 (2.78–4.66)	4.99 (3.98–6.19)	3.51 (2.67–4.53)	5.3 (4.25–6.53)	2.39 (1.71–3.26)
Attended Days at risk	615,821	605,425	613,691	606,784	613,588	605,619	610,508
Non-Attended Admissions (n)	43	28	60	25	46	16	26
Non-Attended Admit Rate (annualised)	2.24 (1.62–3.02)	1.46 (0.97–2.12)	6.41 (4.89–8.24)	2.71 (1.76–4.01)	2.88 (2.11–3.84)	1.01 (0.58–1.64)	1.36 (0.89–1.99)
Non-Attended deaths (n)	9	6	13	15	11	7	8
Non-Attended Death Rate (/100 pt. yrs)	46.6 (21.3–88.4)	31.1 (11.4–67.6)	138.4 (73.7–236.6)	159.9 (89.5–263.8)	68.7 (34.3–123)	44 (17.7–90.6)	41.4 (17.9–81.5)
Non-attended Days at risk	7053	7050	3429	3423	5842	5812	7059

### Hospitalisation and mortality immediately following non-attendance

**Fig. 3:**
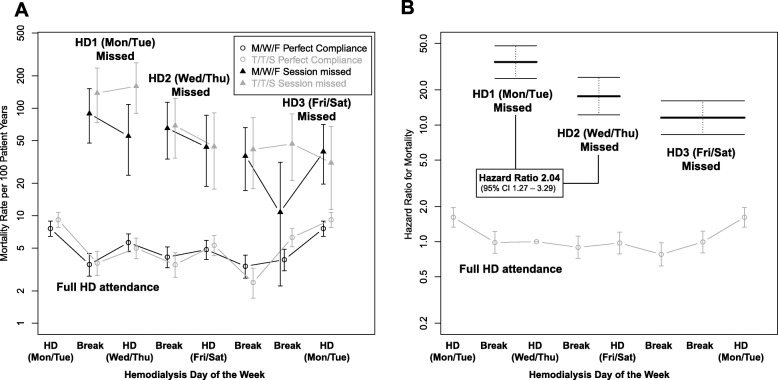
The mortality rate in 100 patient years (**a**) and associated multivariate hazard ratio (**b**) according to dialysis schedule and day of the week for patients who attended and did not attend a HD session. For non-attended sessions, the hazard for mortality is depicted for the days between the missed session and next scheduled session

**Fig. 4:**
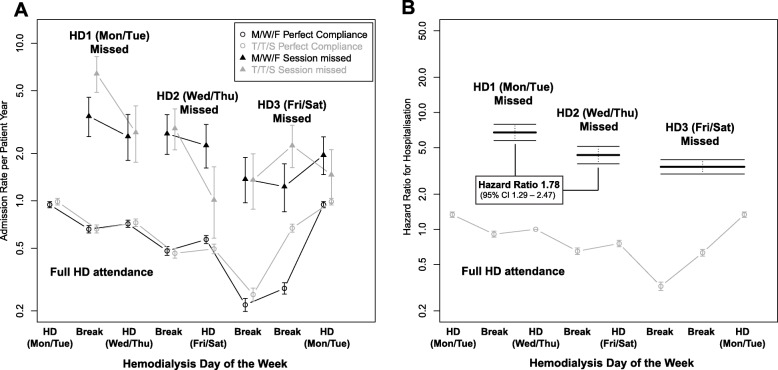
The hospitalisation rate in patient years (**a**) and associated multivariate hazard ratio (**b**) according to dialysis schedule and day of the week for patients who attended and did not attend a HD session. For non-attended sessions, the hazard for mortality is depicted for the days between the missed session and next scheduled session

**Fig. 5:**
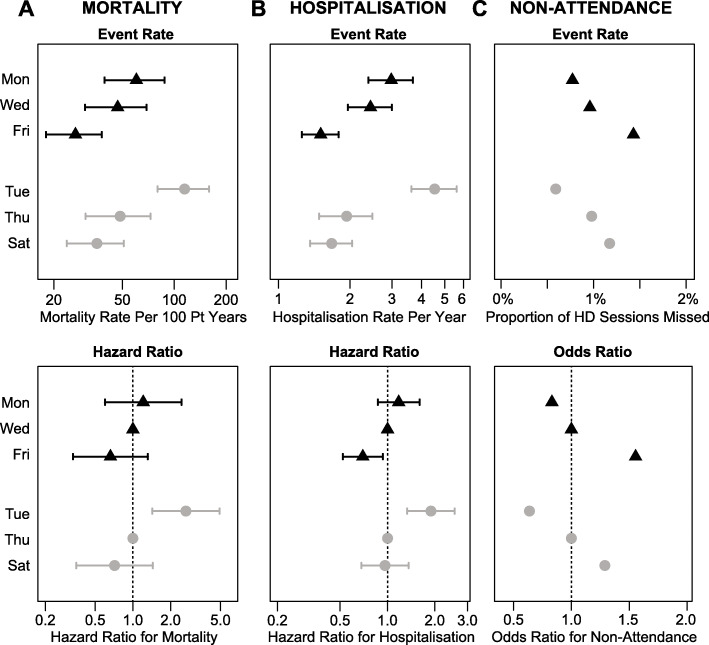
Summary of non-attendance and the subsequent mortality and hospitalisation rates according to dialysis session non-attended. Black: Monday/Wednesday/Friday patients, Grey: Tuesday/Thursday/Saturday patients

Overall, between a missed HD session and the next scheduled HD session, the mortality rate was 51.9 per 100 patient years (95% CI 43.5–61.4), and hospitalisation rate was 2.13 per patient years (95% CI 1.95–2.32), compared to 4.86 (95% CI 4.6–5.1) and 0.58 (95% CI 0.57–0.59) during the same period in those who attended the HD session. The variation in hospitalisation and mortality events and associated event rates, stratified by attendance or non-attendance across the dialysis week is reported in Table 2. These rates were greater if the session missed was the first after the two-day break (Table 2): compared to missing the second session of the dialysis week, the hazard ratio for mortality after missing the first HD and the third HD were 2.04 (95% CI 1.27–3.29) and 0.71 (95% CI 0.44–1.14) respectively. For hospitalisation these were 1.78 (95% CI 1.29–2.47) and 0.92 (95% CI 0.67–1.28) respectively. The variation in these event rates and the multivariate adjusted hazard ratio are shown alongside findings from patients with perfect attendance in Figs. 3 and 4. When performing these analyses on individual schedules, this association failed to meet statistical significance in MWF patients but was evident in TTS patients (Fig. 5a-b).

### Hospitalisation and mortality across the dialysis week in patients who attended their scheduled dialysis session

In patients who attended their scheduled dialysis session and the three preceding HD sessions, mortality was greatest on the day of the first HD session after the long interdialytic interval in both MWF and TTS schedules (Figs. 3 and 4). Mortality rate was 8.3 per 100 patient years after the two-day break compared to 4.9 per 100 patient years for the rest of the week (adjusted hazard ratio 1.71, 95% CI 1.50–1.96). Similarly, hospitalisation in patients who attended their scheduled HD session was greatest in patients after the two-day break (Fig. 4): 0.96 hospitalisations per patient year compared to 0.51 for the rest of the week (adjusted hazard ratio 1.87, 95% CI 1.79–1.95).

### Sensitivity analyses

Our sensitivity analysis which censored on the day a rearranged dialysis session was performed prior to the next scheduled dialysis did not show a significant difference in the hazard of mortality following non-attendance according to which day of the dialysis week was not attended, however hospitalisation in TTS patients after non-attendance for the third session did increase (Figure S2 in Additional File 1). Excluding non-attended dialysis sessions which preceded a change in schedule persisting for two weeks resulted in a reduction in the variation in non-attendance across the dialysis week, however relative to missing the second dialysis session, greater increases in hospitalisation and mortality after missing the first session persisted (Figure S2 in Additional File 1).

## Discussion

There is mounting evidence of the size and mechanism of harm from the two day break in three times a week dialysis. We were able to use session-level data from 9397 patients spanning fifteen countries to show that this harm expands to the period immediately after non-attendance with greater increases in hospitalization and mortality after the long interdialytic interval. Non-attendance was greatest for sessions immediately prior to or scheduled for a weekend day, and even after prior full HD attendance three times a week, there are increases in mortality and hospitalisation endpoints after the long interdialytic interval.

Due to the variation in definitions for adherence in the published literature, the assessment of non-adherence being performed on the period immediately prior to the patient consenting into observational studies introducing a range of biases [4], and the variation in the follow-up period over which the endpoint is assessed, direct comparison between studies is challenging. Non-adherence according to dialysis day of the week, when reported, has varied to a greater extent in TTS patients [5, 16]. The comparable nature of non-attendance between the two schedules may reflect methodological issues, however accounting for changes in HD schedule and rearranged dialysis did not alter the variation in hazard for both our endpoints following non-attendance across the dialysis week. The change in the proportion non-attended on Saturdays in TTS patients following adjustment for rescheduling is logical as a patient may want to attended weekend activities. We found a relative risk of hospitalisation after non-attendance similar to some studies [6], higher than others, and this is likely be due to follow-up starting some time after the non-adherent session and longer follow-up spanning multiple subsequent scheduled sessions [1, 4].

In limiting our analysis to patients with full attendance, we demonstrate how attendance alters mortality and hospitalisation rates. USRDS and the UK Renal Registry data have shown hospitalisation and mortality event rates approximately twice those observed in our study on the first and second HD days [9, 10]. These differences may be due to the methods used to determine the dialysis regimen: The USRDS study determined dialysis schedule by combining the number of dialysis sessions reported per week, with the day of the week the pre-dialysis urea sample was taken [9]. The UK Renal Registry study used individual sessions reported through hospital activity data to assign HD schedules, but due to incomplete dialysis session capture, carried forward the previous schedule when the pattern of attendance deviated [10], and this study shows that ahead of hospitalisation 20.4% of patients deviate from the three times a week schedule. When exploring the two day break effect, absolute differences in event rates comparing the first and second dialysis days across all studies including ours seem comparable.

We cannot determine from these observational data with certainty that missed sessions contribute causally to hospitalisation and dialysis. The relationship could be explained by a confounding factor, such as acute illness which both prevents attendance for scheduled dialysis and leads to hospital admission or death, or that the true timing of death was before the non-attended session. However, it is difficult to explain how these mechanisms which could bias our findings would vary across the dialysis week, since the prevalence or severity of acute illness itself is unlikely to vary to the degree required to explain our observations. This variation could plausibly represent a manifestation of ongoing harm caused by the two-day break manifest beyond the first session of the dialysis week. Our study does not attempt to shed additional light on the likely mechanisms of harm associated with the two-day break, but it does support the conclusion that the long gap is followed by increases in mortality and hospitalisation.

Our study has several notable strengths. The most important being that through utilisation of session level data, we were able to confidently assign and ensure that patients received the scheduled three times a week dialysis for the preceding week in both non-adherent and fully attended analyses. Our analysis makes it unlikely that variation in non-attendance across the dialysis week, with greater non-attendance before the two day break, explains increases in events after the long interdialytic interval. We may also have provided a more accurate measure of the increased risk associated with the long gap than previous studies. Our study is limited by the fact that we have adopted a definition of non-attendance based on the available data, although our rates are similar to those reported by others [6]. To improve our non-attendance data we would have had some objective assessment by dialysis staff as to whether the non-attendance was for medical or logistical reasons [3] or driven by attitudes surrounding the importance of the treatment relative to other events occurring in the patient’s life [2], which may persist post-transplantation and influence graft outcomes [17]. These attitudes may well be informed by the patients’ own beliefs as to what represents a safe interdialytic interval. Like others investigators, we are unable to exclude the fact that non-attendance is the manifestation of death in the preceding 24 h [6], but include these analyses to understand more fully the two day break on mortality. We are confident about our capture of hospitalisations which are similar to recently published rates from other studies [4], although after non-attendance these events are too few for us to look at admission causes and postulate mechanisms.

## Conclusions

From our study, we suggest how a clinician should consider the potential implications of a patient not attending a haemodialysis session should account for which session is missed. Our data also indicate that even following efforts to reschedule dialysis in the 24–48 h after non-attendance, a greater hazard for mortality and hospitalization persist after non-attendance for the HD session after the long interdialytic interval. In fact, the extension of the interdialytic interval from one to two days, even in in patients who attend regularly, is associated with poor outcomes. We hope that our data will help physicians and nurses counselling patients regarding the potential harms of missed dialysis sessions. We believe that with accumulating observational data suggesting clinical harm associated with the long interdialytic interval observational analysis of practices excluding it should inform the design of a clinical trial of dialysis regimens with and without a two-day gap.

## Supplementary information

**Additional file 1.** Country specific non-attendance rates according to session of the dialysis week; Comparison of hazard associated with non-compliance according to dialysis day of the week based on primary analysis, with established changes in dialysis schedules excluded, and censoring at first rearranged dialysis session; statistical justification for summing across days. Figures showing sensitivity analyses outline in main text, methods.

## Data Availability

The datasets generated and/or analysed during the current study are not publicly available due the conditions stated at the time of patient consent and due to the capacity to identify patients, but are available from the corresponding author on reasonable request.

## Abbreviations

ARO: Recognizing Excellence and Optimizing Outcomes
HD: Haemodialysis
MWF: Monday/Wednesday/Friday
TTS: Tuesday/Thursday/Saturday
