# Supplementary material for: Hospitalization and mortality following non-attendance for hemodialysis according to dialysis day of the week: a European cohort study
Source: BMC Nephrol. 2020 Jun 9;21:218. doi: 10.1186/s12882-020-01874-x (PMC7285433; doi:10.1186/s12882-020-01874-x)
Supplement: Supplementary file 1 — Additional file 1. Country specific non-attendance rates according to session of the dialysis week; Comparison of hazard associated with non-compliance according to dialysis day of the week based on primary analysis, with established changes in dialysis schedules excluded, and censoring at first rearranged dialysis session; statistical justification for summing across days. Figures showing sensitivity analyses outline in main text, methods. [file 12882_2020_1874_MOESM1_ESM.docx]

# Supplementary Material: Hospitalization and mortality following non-attendance for hemodialysis according to dialysis day of the week: A European Cohort Study

**Figure S1.** Country specific non-attendance rates according to session of the dialysis week.


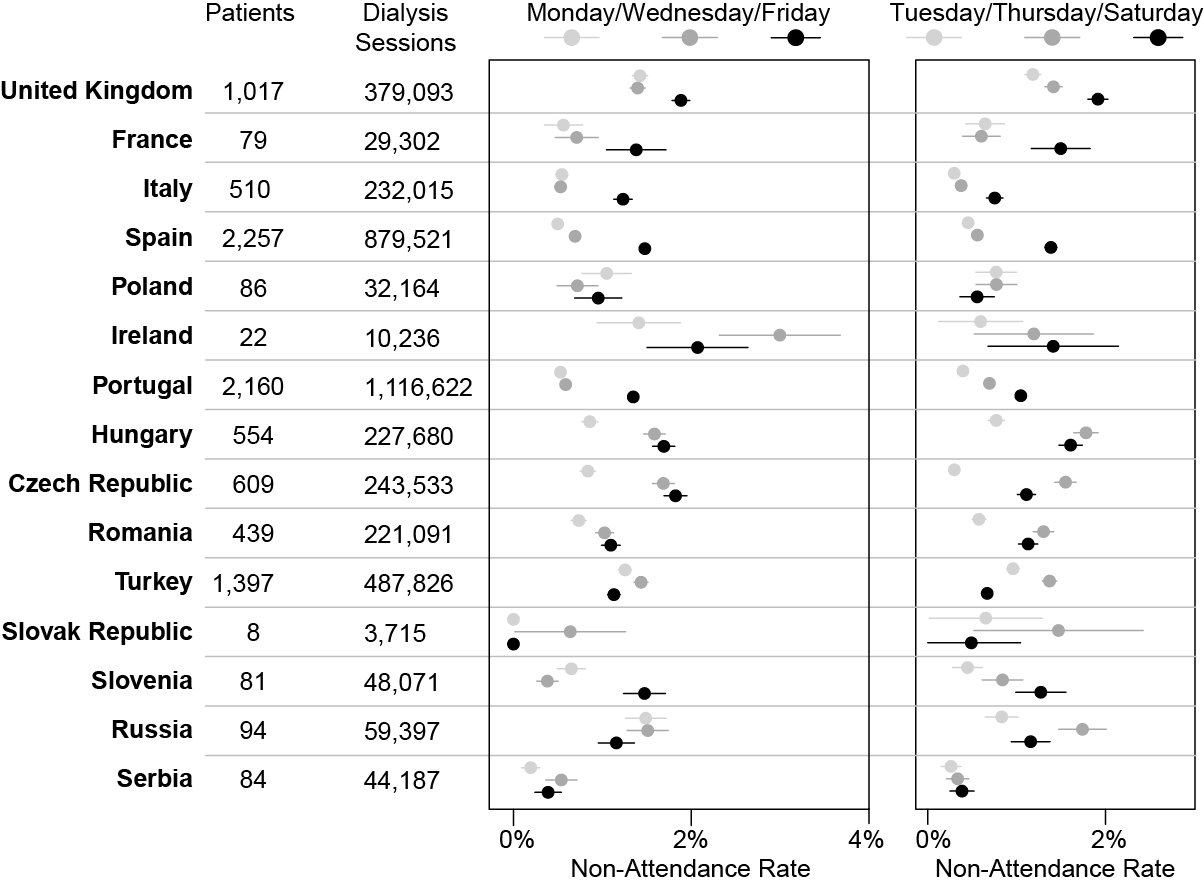


**Figure S2**. Comparison of hazard associated with non-compliance according to dialysis day of the week based on primary analysis, with established changes in dialysis schedules excluded, and censoring at first rearranged dialysis session. Black: Monday/Wednesday/Friday patients, Grey: Tuesday/Thursday/Saturday patients.


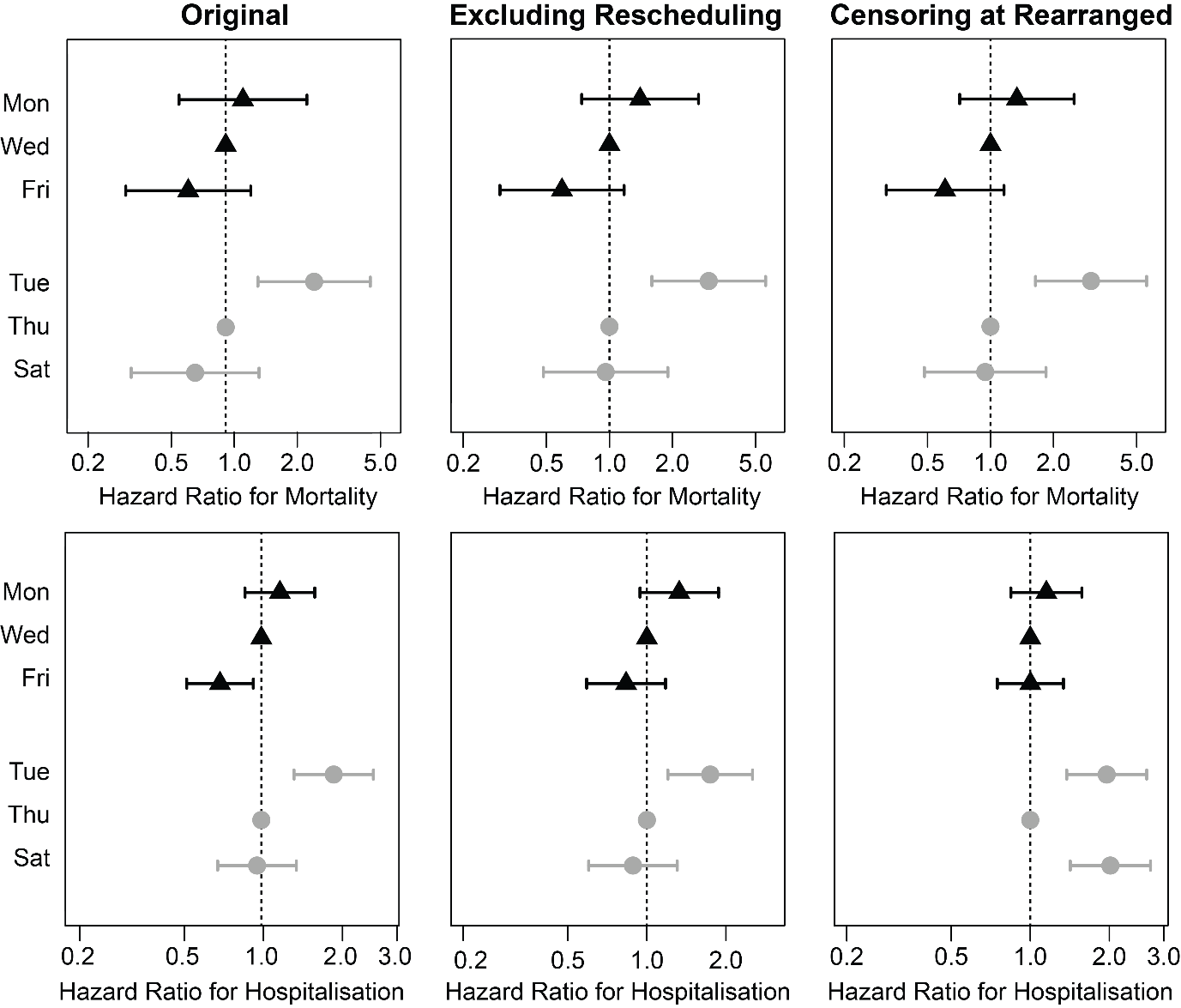


**Risk of dying according to if a patient has attended or not attended for dialysis**

Statistical justification

Let $\lambda_{it}$ be the risk of dying for individual $i$ on day $t$.

Assume a model of the form

$$\eta_{it}=\mu+\alpha_{i}+ \sum_{K=1}^{6} \beta_{k}WD_{k}+ \beta_{7} g(t)$$

$\eta_{it}$ is the linear function using a link function which is either log for a Poisson error function or Logistic for binomial error function. i.e. for a

${WD}_{k}$is a weekday dummy for day k with Wednesday as the baseline.

$g\left( t \right)$ is a function of time

Assume a M/W/F schedule

$g\left( t \right)=1$ if $t$ is a Tue, Thu, Sat and patient did **not** attend day $t, t-1$

$g\left( t \right)=1$ if $t$ is a Wed, Fri, Sun and patient did **not** attend day $t, t-2$

$g\left( t \right)=1$ if $t$ is a Mon and patient did **not** attend day $t, t-3$

$g\left( t \right)=0$ otherwise

For a T/T/S schedule

$g\left( t \right)=1$ if $t$ is a Wed, Fri, Sun and patient did **not** attend day $t, t-1$

$g\left( t \right)=1$ if $t$ is a Thurs, Sat, Mon and patient did **not** attend day $t, t-2$

$g\left( t \right)=1$ if $t$ is a Tue and patient did **not** attend day $t, t-3$

$g\left( t \right)=0$ otherwise

Likelihood

For patient $i$ at time $t$

Let $P_{Nti}=$The probability of dying when not at risk (ie $g\left( t \right)=0$)

$P_{Rti}=$The probability of dying when at risk (ie $g\left( t \right)= 1$)

Assume, for present, $\alpha_{i}$ is constant and no weekday effect from model

Ie. $y_{it}=\mu+\beta g\left( t \right)$

Using a log link $\log\left( P_{N} \right)= \mu$

$\log\left( P_{R} \right)= \mu+ \beta$

Let $N_{i}$ be the number of days the patient is observed

Let $N_{Ri}= \sum_{t=1}^{N_{i}} g (t) , N_{Ni}= N_{i}-N_{Ri}$

$N_{Ri}$ is the number of exposed days for patient $i$

$N_{Ni}$ is the number of not exposed days for patient $i$

If patient $i$ dies on a day not exposed, probability of event is proportional to

$$l_{i}= {P_{N}(1-P_{N})}^{N_{Ni-1}}{(1-P_{R})}^{N_{Ri}}$$

i.e. they die on one day, they don’t die on $N_{Ni-1}$other not exposed days and $N_{Ni}$ exposed days.

If a patient $i$ dies on an exposed day

$$l_{i}= {P_{N}(1-P_{N})}^{N_{Ni}}{(1-P_{R})}^{N_{Ri}-1}$$

If a patient $i$ does not die on an exposed day

$$l_{i}= {P_{N}(1-P_{N})}^{N_{Ni}}{(1-P_{R})}^{N_{Ri}}$$

Let $D_{R}$ number of deaths on an exposed day

$D_{N}$ number of deaths on a not exposed day

${D=D}_{R}+D_{N}$ $N=$Number of patients

Then log likelihood is

$$\log l= \sum_{i=1}^{D_{N}} \log P_{N}+\left( N_{Ni}-1 \right)\log\left( 1-P_{N} \right)+log(1-P_{R})$$

$$+ \sum_{i=D_{N}+1}^{D_{N}+D_{R}} \log P_{R}+\left( N_{Ni} \right)\log\left( 1-P_{N} \right)+\left( N_{Ri}-1 \right)log(1-P_{R})$$

$$+ \sum_{i=D_{N}+D_{R}+1}^{N} N_{Ni}\log\left( 1-P_{N} \right)+N_{Ri}log(1-P_{R}$$

If you differentiate with respect to $P_{N}$and $P_{R}$ and equate to zero you find after a bit of algebra

$$P_{N}= \frac{D_{N}}{\sum_{i=1}^{N} N_{Ni}} and = \frac{D_{R}}{\sum_{i=1}^{N} N_{Ri}}$$

i.e. The risk of dying for someone not exposed is the number of deaths in the not exposed divided by the number of days not exposed and similarly for the number in the exposed group
